# Confinement Effect on Porosity and Permeability of Shales

**DOI:** 10.1038/s41598-019-56885-y

**Published:** 2020-01-08

**Authors:** Jan Goral, Palash Panja, Milind Deo, Matthew Andrew, Sven Linden, Jens-Oliver Schwarz, Andreas Wiegmann

**Affiliations:** 1 Department of Chemical Engineering, University of Utah, Salt Lake City, Utah USA; 2 Energy & Geoscience Institute, University of Utah, Salt Lake City, Utah USA; 3 Carl Zeiss X-Ray Microscopy, Pleasanton, California USA; 4 Math2Market GmbH, Kaiserslautern, Germany

**Keywords:** Petrology, Crude oil, Natural gas, Imaging techniques, Scanning electron microscopy

## Abstract

Porosity and permeability are the key factors in assessing the hydrocarbon productivity of unconventional (shale) reservoirs, which are complex in nature due to their heterogeneous mineralogy and poorly connected nano- and micro-pore systems. Experimental efforts to measure these petrophysical properties posse many limitations, because they often take weeks to complete and are difficult to reproduce. Alternatively, numerical simulations can be conducted in digital rock 3D models reconstructed from image datasets acquired via e.g., nanoscale-resolution focused ion beam–scanning electron microscopy (FIB-SEM) nano-tomography. In this study, impact of reservoir confinement (stress) on porosity and permeability of shales was investigated using two digital rock 3D models, which represented nanoporous organic/mineral microstructure of the Marcellus Shale. Five stress scenarios were simulated for different depths (2,000–6,000 feet) within the production interval of a typical oil/gas reservoir within the Marcellus Shale play. Porosity and permeability of the pre- and post-compression digital rock 3D models were calculated and compared. A minimal effect of stress on porosity and permeability was observed in both 3D models. These results have direct implications in determining the oil-/gas-in-place and assessing the production potential of a shale reservoir under various stress conditions.

## Introduction

Conventionally, oil and gas have been recovered from sandstone or carbonate reservoirs where hydrocarbons are trapped in well-connected systems of pores and fractures. Thanks to recent advancements in petroleum technologies, such as horizontal drilling and hydraulic fracturing, oil and gas can also be recovered unconventionally from less-developed mudstone (shale) reservoirs – deep and tight rock formations of heterogeneous lithology and mineralogy with poorly-connected nanometer-/micrometer-size pore systems. Among the key factors in assessing the oil/gas productivity potential of shale reservoirs (also referred as to shales) are the porosity and permeability of these oil- and/or gas-bearing rock formations. Porosity of a rock is the volume of void space, which can be filled with different reservoir fluids (e.g., oil, gas, water) at various saturations, whereas permeability of a rock is the ability of these fluids to flow within and between the pore space. Shales are characterized by very low porosity (typically less than 5%) and very low permeability (typically less than 1,000 nD), which make them challenging in recovering economically viable hydrocarbons. Determining the volume of oil and/or gas present in a reservoir (oil- and/or gas-in-place), and its potential to flow through reservoir pore/fracture system into the wellbore, helps petroleum industry to understand and optimize the producibility of a reservoir.

Porosity and permeability of shales are often determined by examining core rock samples recovered from oil/gas wells drilled deep into rock formation. Recently, modern 2D/3D imaging techniques, have been used to investigate mineralogy and porosity in very fine detail, down to the sub-nanometer level^1–4^. These methods (and advanced image analysis) have facilitated characterization of the pore morphology within both the organic matter and non-organic (mineral) matrix of shales^5–14^. However, there is a little debate whether this imaging data of recovered samples (from thousands of meters) can be considered representative of an unstressed rock.

Therefore, the objective of this study was to investigate the effect of reservoir confinement on porosity and permeability of an organic-rich Marcellus Shale rock sample imaged with FIB-SEM nano-tomography at ultra-high-resolution (5 nm/voxel). Porosity and (absolute) permeability under non-confined and confined conditions (at different reservoir depths) were simulated and compared by compressing two digital rock 3D models and re-evaluating the above petrophysical properties.

## Background

Researchers are increasingly focusing on modeling and simulation of fluid flow and transport phenomena in shales in order to measure their permeability due to the inherent difficulty of conducting core flooding experiments in these extremely tight rocks. Even under high pressure differential (1,000–5,000 psi), it takes several days for different fluids (e.g., decane, methane, water, brine) to pass through nano-/micro-pore systems of a core rock sample^15–17^.

Numerical simulations are one of the best alternative methods circumventing these problems. Contrary to analytical and semi-analytical techniques, numerical methods are capable of handling complex models of shales and simulating single-/multi-phase flow of various fluids in these nanoporous rocks^18–21^. Depending on the complexity of the model and/or the objectives of the studied problem, the scale and features of the simulated model may vary from nanometers to centimeters. Various computational models, such as molecular dynamics (MD)^22,23^, direct simulation Monte Carlo (DSMC)^24^, dissipative particle dynamics (DPD)^25,26^, Lattice Boltzmann (LB)^27,28^, and many continuum-flow-based models, have been used for simulations of fluid flow in shales to calculate their permeability. The main deference between these simulations lies in the size of the modeled system and the type of fluid flow they are capable of modeling. It is well established that different fluid flow and transport phenomena occur in shales (e.g., Darcy’s flow in macro-pores, non-Darcy’s flow in nano-pores, or adsorption/absorption of the organic or mineral matrix)^29–32^. Thus, fluids (oil, gas, water/brine, or a combination of these three) can be carried by means of multiple transport mechanism – from continuum to free-molecular flow. However, none of the above-mentioned computational models are capable of capturing all of these mechanisms and performing fluid flow simulations in realistic 3D models, reflecting actual nanoporous microstructure of shales.

Various parameters, such as heterogeneous mineralogy, porosity (pore type/size and structure), or rock compaction with depth, may impact permeability of shales^33,34^. Therefore, it is critical to take all of these factors into consideration while modeling these nanoporous rocks, and performing fluid flow simulations in their intricate nano- and micro-pore systems. Therefore, in order to investigate the effect of reservoir confinement on porosity and permeability of shales, in this study, five consecutive mechanics (compression) and fluid flow (permeability) simulation scenarios (reflecting actual shale reservoir conditions) were performed on two realistic digital rock 3D models reconstructed from nanoscale-resolution FIB-SEM nano-tomography image datasets of a rock sample collected from the Marcellus Shale – one of the most prolific shale plays in the United States^35,36^.

## Results and Discussion

**Figure 1:**
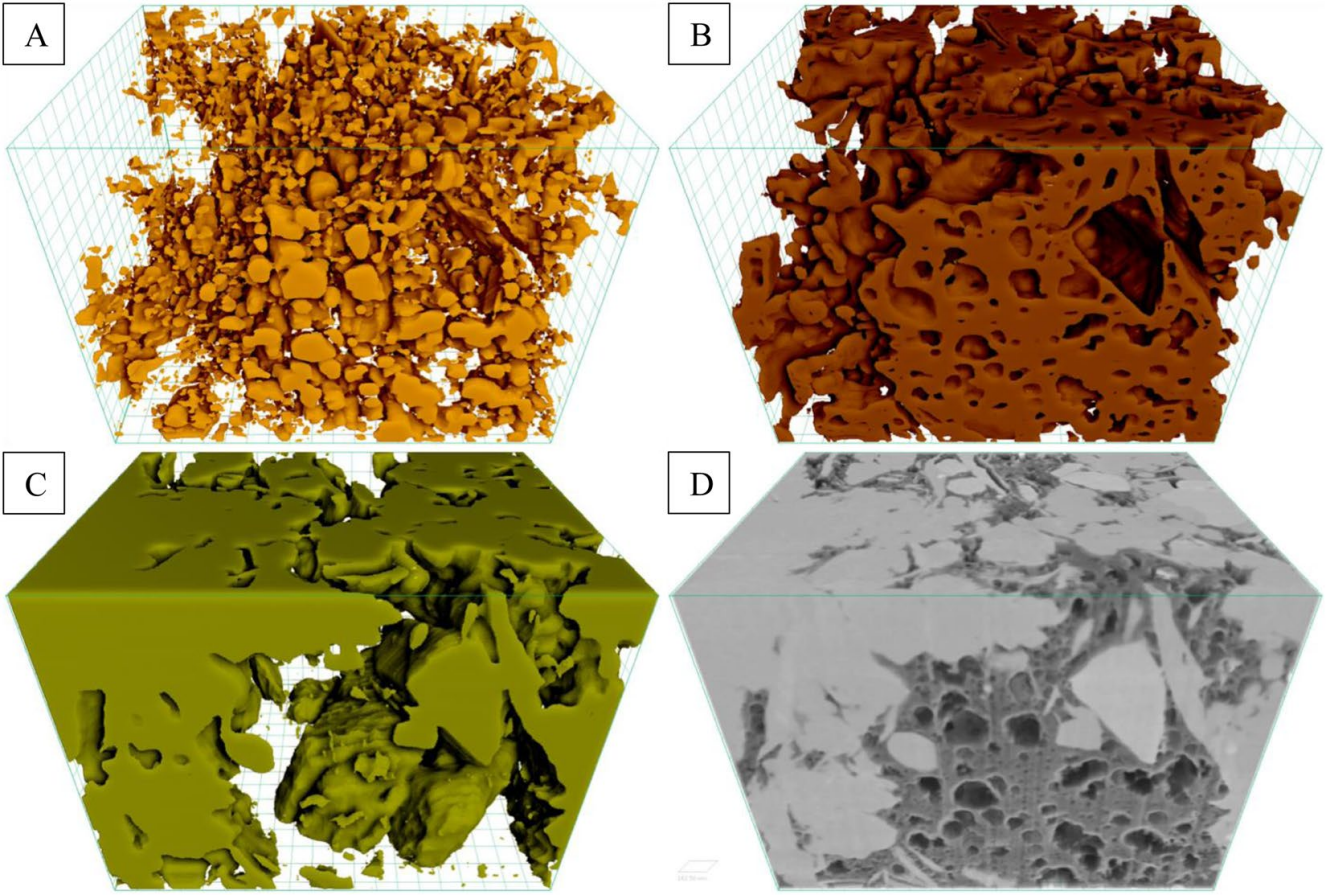
Visualization of segmented (**A**) pores, (**B**) organic matter, and (**C**) mineral phases present within digital rock 3D model reconstructed from (**D**) FIB-SEM nano-tomography image dataset of the ROI-1.

**Figure 2:**
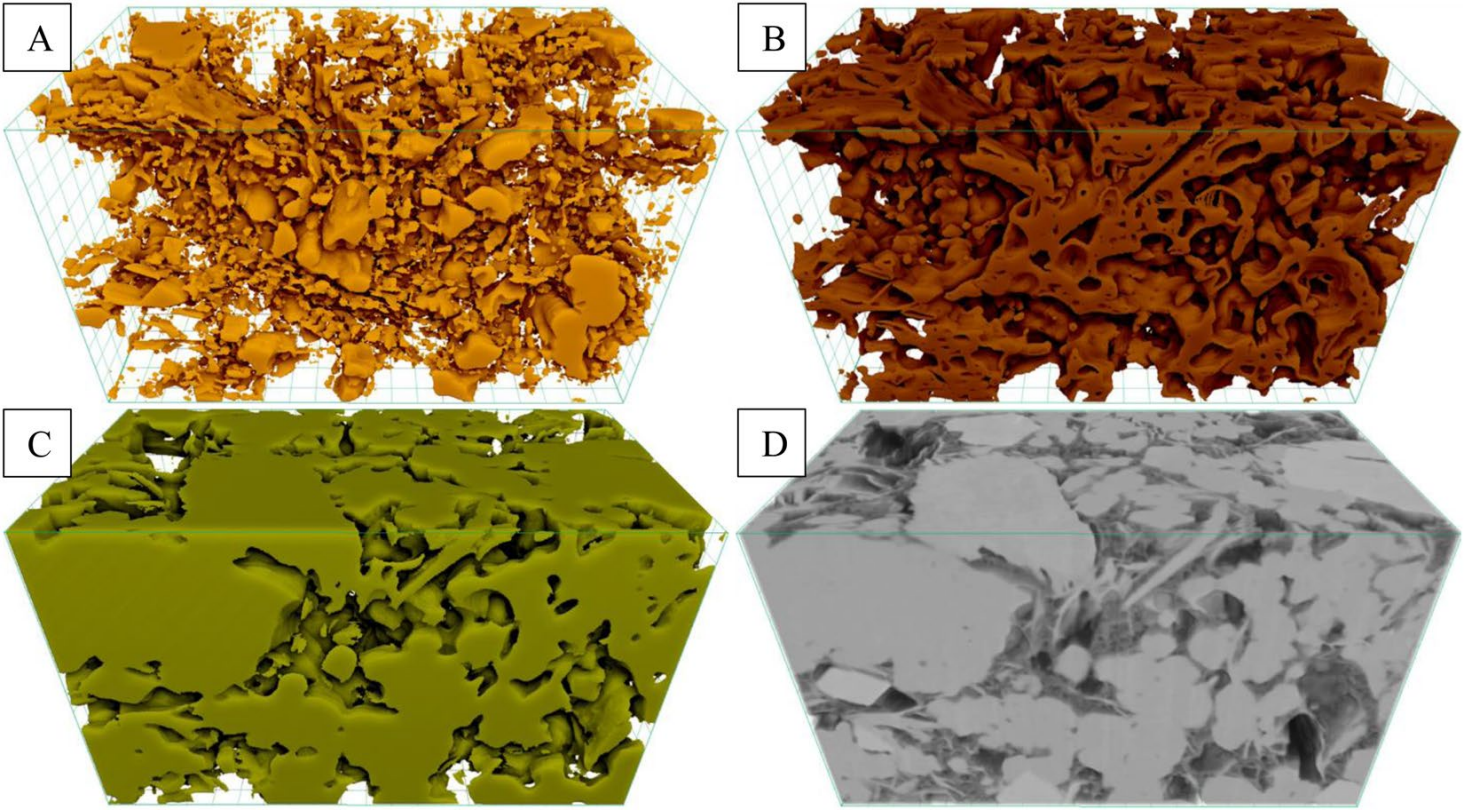
Visualization of segmented (**A**) pores, (**B**) organic matter, and (**C**) mineral phases present within digital rock 3D model reconstructed from (**D**) FIB-SEM nano-tomography image dataset of the ROI-2.

Two digital rock 3D models, reconstructed from 5 nm/voxel-resolution FIB-SEM nano-tomography image datasets of two regions on interest (ROIs) of a Marcellus Shale rock sample (ROI-1 and ROI-2), are shown in Figs. 1 and 2. The volume fractions of pores, organic, and mineral phases present within both of these 3D models are presented in Table 1. As shown in the Table 1, the ROI-1 had higher organic matter content and lower mineral matter content than the ROI-2.

**Table 1:** Volume fractions of pore, organic and mineral phases present within digital rock 3D models reconstructed from FIB-SEM nano-tomography image datasets of the ROI-1 and ROI-2.

3D Model	Pore Phase	Organic Phase	Mineral Phase
ROI-1	13.2%	38.6%	48.2%
ROI-2	14.7%	25.2%	60.1%

**Figure 3:**
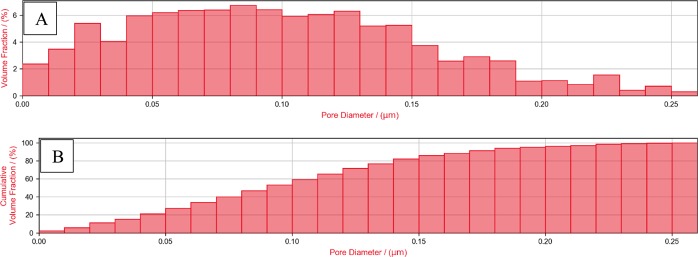
(**A**) Relative and (**B**) cumulative pore size distribution (PSD) of the ROI-1 3D model.

**Figure 4:**
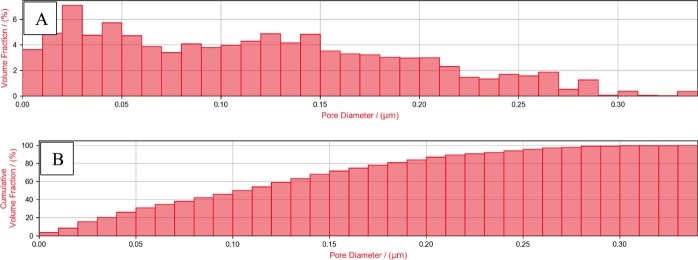
(**A**) Relative and (**B**) cumulative pore size distribution (PSD) of the ROI-2 3D model.

Pore size distribution (PSD) analysis of the investigated digital rock 3D models showed pores of similar (but slightly different) sizes within both regions. The PSD of the ROI-1 and ROI-2 are shown in Figs. 3a and 4a respectively. The diameter of the pores ranged from approximately 5 nm to 350 nm, where half of all the pores had diameter smaller than 90 nm (for ROI-1) and 110 nm (for ROI-2), as shown in cumulative PSD in Figs. 3b and 4b.

Next, ROI-1 and ROI-2 3D models were used as an input for compression simulations, which reflected five different reservoir confinement scenarios: 2,000 ft to 6,000 ft depth – production interval of a typical oil/gas reservoir within the Marcellus Shale play^37^. The reservoir confinement (stress), which acted at each of the two 3D models at five different depths (used as an input in compression simulations) are presented in Table 2. Geomechanical properties, such as Young’s modulus (*E*) and Poisson’s ratio (υ), taken from Bennett *et al*. (2015)^38^, were assigned both to the organic (*E*: 7.65 GPa at 0.3 υ) and mineral phases (*E*: 23.15 GPa at 0.3 υ) of the 3D models. After each of the compression simulations, porosity and permeability of the deformed geometries were reexamined.

**Table 2:** Compression simulation set-up parameters: vertical stress (*σ*_*v*_) = 1 psi/ft and horizontal stress (${\sigma }_{{H}_{min/max}}$) = 0.6 psi/ft.

Scenario	Depth [ft]	σ_*v*_ [psi]	${{\boldsymbol{\sigma }}}_{{{\boldsymbol{H}}}_{{\boldsymbol{\min }}}}$[psi]	${{\boldsymbol{\sigma }}}_{{{\boldsymbol{H}}}_{{\boldsymbol{\max }}}}$[psi]
1	2,000	2,000	1,200	1,200
2	3,000	3,000	1,800	1,800
3	4,000	4,000	2,400	2,400
4	5,000	5,000	3,000	3,000
5	6,000	6,000	3,600	3,600

### Pre- and post-compression porosity analysis

**Figure 5:**
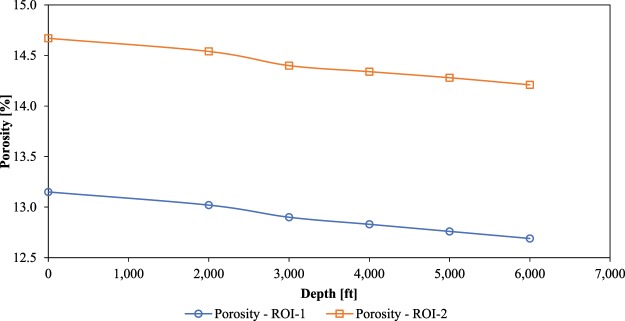
Porosity of the ROI-1 and ROI-2 3D models after five compression simulations.

Porosity of the non-compressed 3D models of the ROI-1 and ROI-2 were 13.2% and 14.7% respectively. These porosities were then re-evaluated after each of the compression simulations. Figure 5 shows porosity results as a function of depth for both, the ROI-1 and ROI-2 3D models. The porosity of both 3D models decreased, however not that significantly. A maximum change of 3.5% (for the ROI-1) and 3.1% (for the ROI-2) in porosity (in relation to their initial porosities), at the maximum depth of 6,000 ft, was observed.

It is evident from the results that the shale rock is compacted under the stress and the pore space is reduced consequently. The amount of reduction depends on the combined effect of stress and the overall geomechanical properties that are a function of the contribution from organic and mineral phases. It is clear from the values of Young’s modulus that the organic phase (*E*: 7.65 GPa) is more deformative than mineral phase (*E*: 23.15 GPa). Since the ROI-1 3D model had higher organic matter content than the ROI-2, it experienced higher porosity reduction under the same stress.

### Pre- and post-compression permeability analysis

Permeability of the non-compressed 3D model of the ROI-1 was calculated to be 95.6 nD (in the Z-direction) and 5.3 nD (in the Y-direction), whereas permeability of the ROI-2 was 3.6 nD and 1.4 nD in the Z- and Y-directions respectively. There was no flow (permeability) recorded in the X-direction in both 3D models. It is very common for shales, that their permeability changes with the direction of flow. This is due to the complex geometry of their pore systems, which is not uniform in all directions (anisotropic in nature). It was also noticed, that the ROI-1 3D model had much higher permeability than the ROI-2 3D model. This was due to higher interconnectivity of its pore systems.

**Figure 6:**
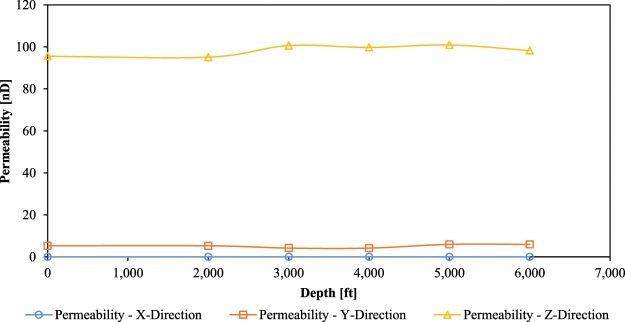
Permeabilities of the ROI-1 3D model after five compression simulations.

**Figure 7:**
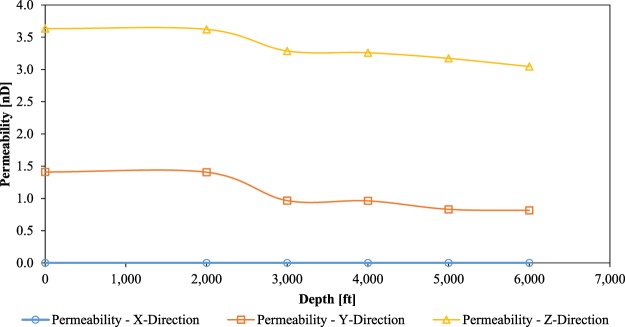
Permeabilities of the ROI-2 3D model after five compression simulations.

Permeabilities of the ROI-1 and ROI-2 3D models, after five compression simulations, are plotted in Figs. 6 and 7 respectively. The permeability of the ROI-1 increased by 12.3% in the Y-direction and 2.7% in the Z-direction (in relation to its initial permeability). On the other hand, permeability of the ROI-2 decreased by 42.6% in the Y-direction and 16.3% in the Z-direction (also in relation to its initial permeability). The permeability enhancement of the ROI-1 is somewhat counter-intuitive. Normally, it is expected from compressed rocks to become less permeable (like in the case of the ROI-2). In order to investigate these phenomena, fluid flow pathways (percolation paths) of the pre- and post-compression 3D models were examined.

**Figure 8:**
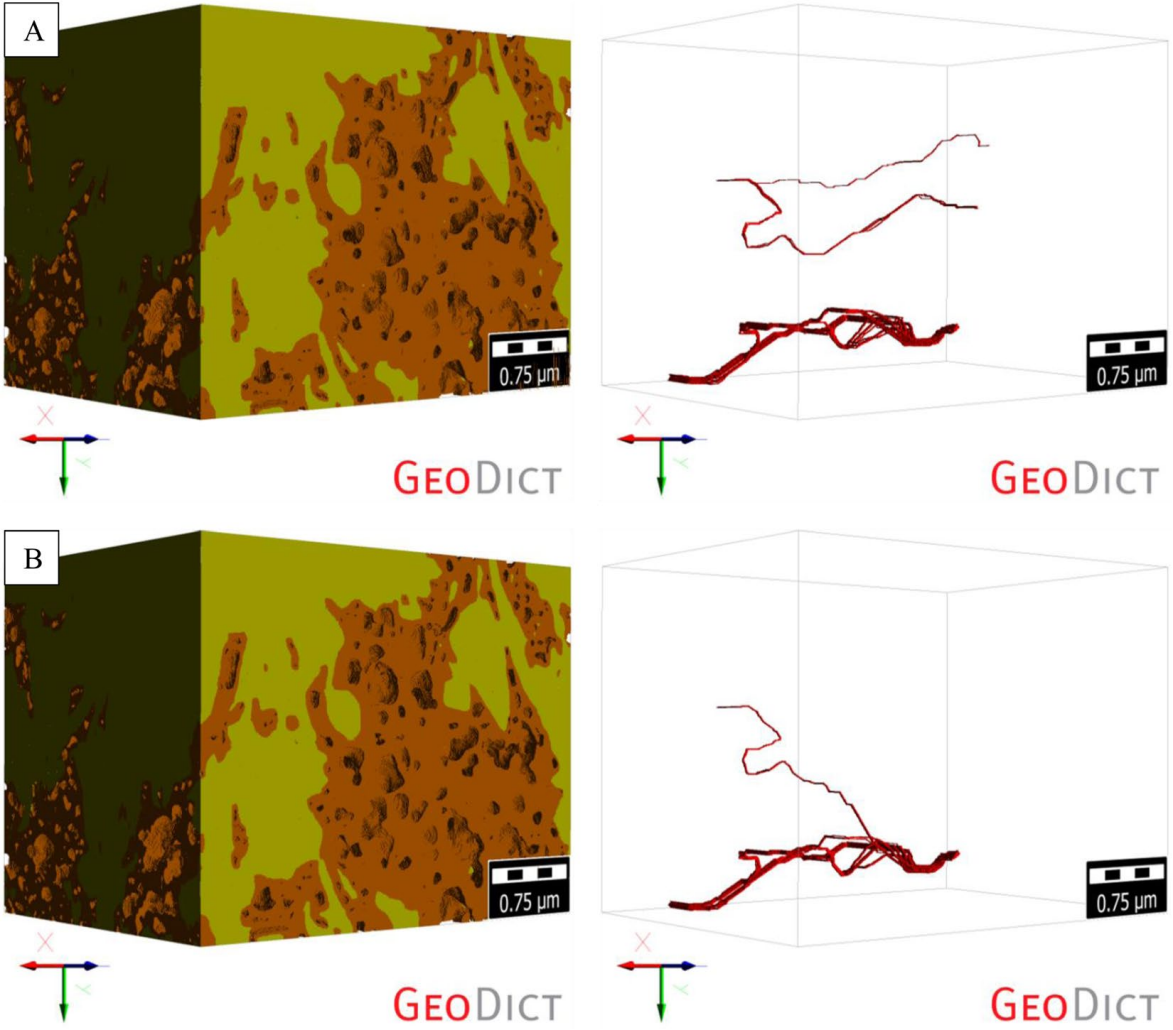
Visualization of the percolation paths of the (**A**) non-compressed and (**B**) compressed (at the maximum depth of 6,000 ft) ROI-1.

The percolation path analysis of the first non-compressed digital rock 3D model (ROI-1), and its preferential fluid flow pathways in the Z-direction, showed a total of 23 percolation paths (Fig. 8a). The length of the largest path was calculated and equal to 3.799 um, and the diameter of the largest particle that could be transported through the non-compressed ROI-1 was equal to 11.18 nm. The same analysis of the compressed ROI-1 showed a significant reduction in the total number of percolation paths to 12, with the largest path length of 3.769 um – slight decrease (Fig. 8b). It also showed that the maximum particle diameter (that could be transported through the compressed ROI-1) actually increased to 15 nm. Fewer pathways which were slightly larger on the average led to a small increase in permeability for this first ROI-1. These phenomena explain a slight increase in the permeability of the first digital rock 3D model after compression.

**Figure 9:**
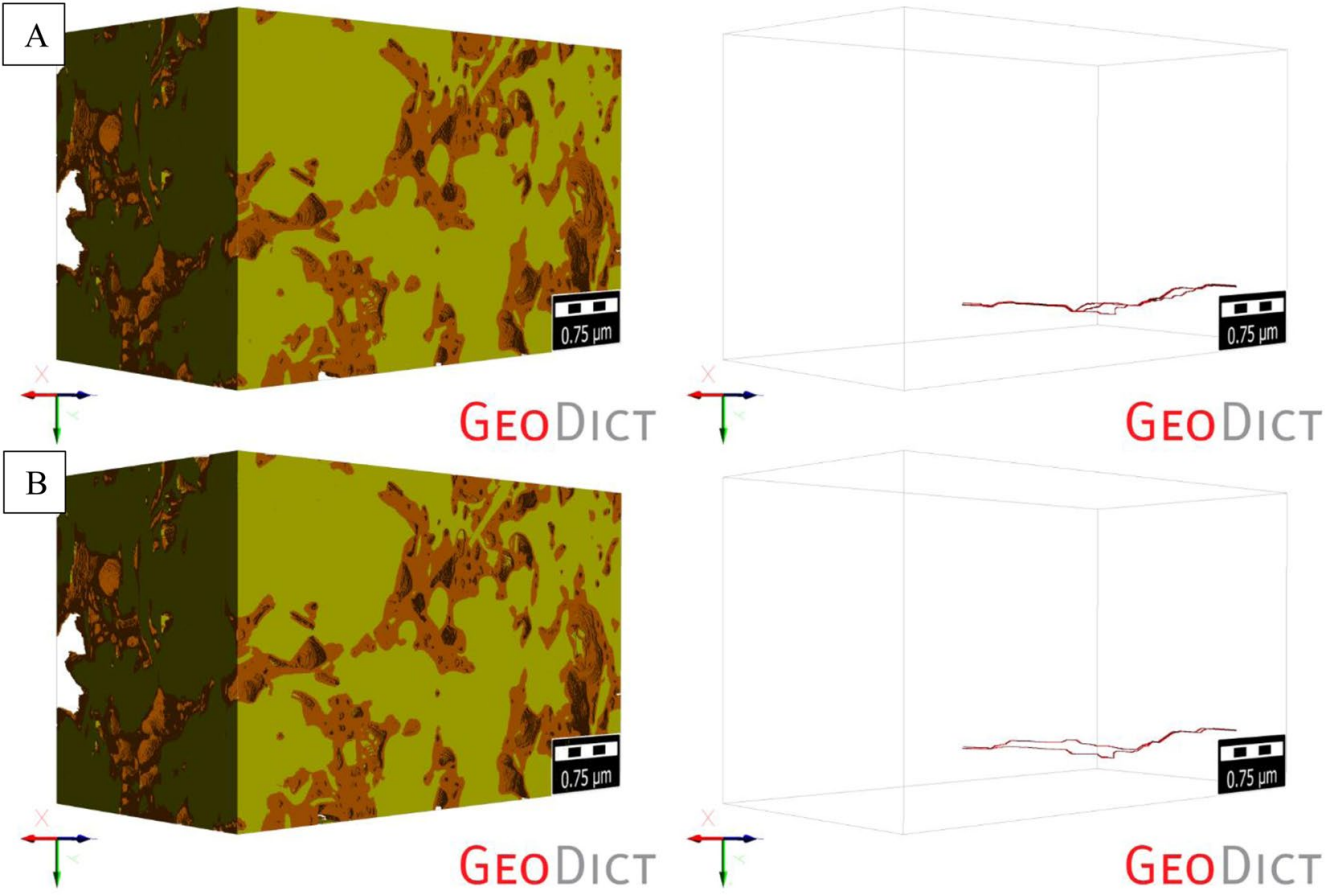
Visualization of the percolation paths of the (**A**) non-compressed and (**B**) compressed (at the maximum depth of 6,000 ft) ROI-2.

A similar analysis was performed on the second digital rock 3D model (ROI-2). The analysis showed a total of 3 and 2 percolation paths for the non-compressed (Fig. 9a) and compressed ROI-2 (Fig. 9b), respectively. The maximum path length was calculated and equal to 2.666 um (for the non-compressed ROI-2) and 2.643 um (for the compressed ROI-2). The maximum particle diameter that could be transported through both, non-compressed and compressed ROI-2, was equal to 5 nm and remained the same.

The percolation analysis also highlighted the calculated permeability differences between the two ROIs. ROI-1 with larger number of relatively wider pathways led to a permeability of around 100 nD, whereas the limited number of narrow fluid flow pathways restricted the permeability of the ROI-2 to single-digit nD range.

The results showed that while dealing with highly-heterogenous porous media, such as shales, and their irregular pore structures, shale pore morphological characteristics (e.g., percolation paths) may differ from sample to sample (region to region) and may change when subjected to confinement in a manner that is not necessarily similar to that of homogenous materials. This pressure change may affect pore geometries (and hence percolation paths) affecting shale porosity and permeability to certain extent.

Although the porosity and permeability modeling and simulation results may not be representative of the entire feet-long core rock sample, nor miles-long shale reservoir, they show that reservoir confinement effect on these petrophysical properties is minimal, and hence porosity and permeability of shales should remain almost the same regardless of recovering these rocks deep from the subsurface.

## Methods

Traditional core analytical techniques commonly used to determine porosity or permeability of oil-/gas-bearing rocks, developed for conventional (sandstone or carbonate) reservoirs, are limited in terms of characterizing unconventional (shale) reservoirs. This adds to the difficulties of exploration intended for production which requires a search for a new solution to supplement the existing characterization methods. Petroleum industry is now turning to the technique of “digital rock” as a potential solution for characterizing shales, owing to the power of modern microscopes to reliably and precisely image and analyze these rocks at the micro- and/or nanoscale-resolution. Digital rock analysis (also known as digital rock physics) integrates multi-scale and multi-modal 2D/3D imaging techniques (e.g., field-emission scanning electron microscopy (FE-SEM) or focused ion beam – scanning electron microscopy (FIB-SEM) nano-tomography) together with advanced image analysis methods. These digital rock 3D models can be then used to investigate e.g., nanoporous microstructure of geomaterials in very fine detail, or for modeling and simulation of various multi-physics processes that take place within these geosystems deep in the subsurface.

### Nanoscale-resolution 3D imaging with focused ion beam–scanning electron microscopy (FIB-SEM) nano-tomography

FIB-SEM nano-tomography (serial-sectioning) is a nanoscale-resolution 3D imaging technique in which cross-section ion milling is used to controllably remove approximately 5- to 20-nm-thin layer of material (“slice”) of the sample, and electron imaging is used to characterize the freshly prepared sample surface. Automated sequential FIB milling and SEM imaging allows for the acquisition of a series of images, which in turn leads to digital rock 3D model reconstruction.

In this study, a ZEISS Crossbeam 550 FIB-SEM was used to collect ultra-high-resolution (5 nm/voxel) image datasets of two organic-rich regions of interest (ROIs) of a Marcellus Shale rock sample. Both, secondary electron (SE) and backscatter electron (BSE) signals were acquired at the time of the 3D imaging. These SE and BSE images were then blended into a single image dataset to optimize brightness and contrast between organic, mineral, and pore phases.

### Image analysis: image processing and (machine learning) image segmentation

Image processing was first performed in order to improve image quality – a combination of different filters and operations was applied in order to remove imaging artifacts, noise, and other background intensity variations from the images. Commonly found artifacts in FIB-SEM nano-tomography image datasets are e.g., curtaining or shadowing effect. These can be removed by applying various image processing algorithms (e.g., FFT, local or non-local means filters). Image segmentation, on the other hand, allows for classification of the images into segments representing different (shale rock) material components (of different densities, and hence of different intensities in the images). In this study, machine learning segmentation was applied in order to segment pore, organic, and non-organic (mineral) phases within the FIB-SEM nano-tomography image datasets.

In the past decade, machine learning grew out of the quest for artificial intelligence and has found application in many areas (e.g., self-driving cars, effective web search), however its application to image analysis has been recently developed. Machine learning algorithms enable it to identify patterns in observed data and build predictive models without being explicitly programmed. Machine learning image segmentation automatically partition challenging image datasets, that may carry a variety of modality-specific imaging artifacts, into segments (labels), representing different groups of features of the rock microstructure (e.g., pores or minerals), previously too difficult to segment by threshold- or watershed-based approaches^39^.

Machine learning image segmentation was performed with the ZEN Intellesis software, developed by ZEISS, which uses open-source packages (such as scikit-image and SciPy) for feature extraction. The machine learning capability allows for interactive training of the microscopy 2D/3D images. A combination of different filters (e.g., mean, Gaussian, Gabor, Hessian, Sobel) are used to extract relevant features from each greyscale channel of an image. A machine learning algorithm – forest of randomized trees – is used to train the segmentation model (classifier). The classifier is then applied to a slice displayed or a 3D volume. This process is iterative where additional data may be added to refine the segmentation of a 2D slice or a 3D volume. The final segmented 2D/3D image is produced by using the satisfactorily-trained segmentation model to the entire image dataset^40^.

The processed and segmented image datasets were then reconstructed into two digital rock 3D models: ROI-1 (3.25 µm × 2.5 µm × 2.5 µm) and ROI-2 (3.75 µm × 2.25 µm × 2 µm). These two 3D models were then used for mechanics (compression) and fluid flow (permeability) simulations performed using the GeoDict software. The GeoDict software, developed by Math2Market, brings different solution methods into play for the simulation of mechanics and single-phase flow in porous media^41^. The common denominator of the computational models (solvers), used in this software, is that they work directly on 3D models. They do not require a mesh generation step, which is a bottleneck for classical finite-element or finite-volume methods.

### Mechanics (compression) simulations

For a uniform macroscopic strain *S*, the boundary value problem (BVP) for the stress field *σ*, strain field *ε*, and displacement field μ* can be stated. The BVP or equations of linear elasticity consists of the elastic equilibrium Eq. (1), Hooke’s law (2), and periodic boundary conditions (3):

1 $$\nabla \cdot \sigma =0$$

2 $$\sigma =C:{\epsilon }$$

3 $$2{\epsilon }=2S+\nabla {u}^{\ast }+{(\nabla {u}^{\ast })}^{T}$$

By introducing a reference material of homogeneous stiffness *C*_0_., the BVP can be transformed into the strain-based Lippmann-Schwinger Eq. (4):

4 $$(I+{B}_{{\epsilon }}){\epsilon }=\epsilon +{\Gamma }_{0}\ast ((C-{C}_{0}):{\epsilon })=E$$

Fast Fourier transform (FFT) allows to solve the convolution wh the Green’s operator Γ_0_^42^. The Lippmann-Schwinger equation can also bformulated with respect of stress instead of strain. These equations can iteratively be solved using the Neumann series expansion, the so-called “basis scheme”. Instead of using the Nmann series expansion, Krylov subspace methods can be applied to accelerate the convergence of the method. The formulation allows to handle linear and non-linear (i.e., replacing Hooke’s law with a non-linear formulation) material laws as well as isotropic, transverse-isotropic, orthotropic, or anisotropic constituent materials.

Similar to the fluid flow solvers (described below), a staggered grid is used to discretize displacement, strain, and stress variables^43^.

For the computation of compression dependent properties, the displacement field μ*. used to predict a compressed structure. The voxels of the original image are moved along the displacement field and cut with a reduced voxel image. The result of that procedure is a grayscale image, where a global threshold is used to perform a segmentation of the different phases. The threshold is chosen in the FeelMath solver such that either mass or volume is preserved.

### Fluid flow (permeability) simulations

A laminar flow of aluid with constant viscosity *μ*, velocity *μ*, and pressure *p* are described by Stokes conservation of momentum (5), conservation of mass (6), and no-slip boundary conditions (7). The domain Ω is periodic and the direction of the flow is induced by the unit vector *f*. that always points in the direction of one of the coordinate axes:

5 $${\rm{\mu }}{\nabla }^{2}{\rm{u}}-\nabla {\rm{p}}={f}$$

6 $$\nabla \cdot u=0$$

7 $$u{|}_{\partial \Omega }=0$$

The no-slip boundary conditions (7) are applicable forlow movement of fluid in the porous media. Following the set of Eqs. (5, 6, and 7), a few fluid flow simulations are conducted in the digital rock 3D models to calculate effective material properties, such as permeability. In these simulations, the mean flow velocity is measured for an applied pressure drop across the porous media. Although the Eqs. 5, 6, and 7) do not contain permeability (*K*) term, it is calculated during post-processing step. The relationship between the predicted mean flow velocity for a pressure drop in a porous media is expressed by a constitutive equation, commonly-known as Darcy’s law (8):

8 $$u=-\,\frac{K}{{\rm{\mu }}}(\nabla {\rm{p}}-f)$$

where *K* is the permeability of the material.

**Figure 10:**
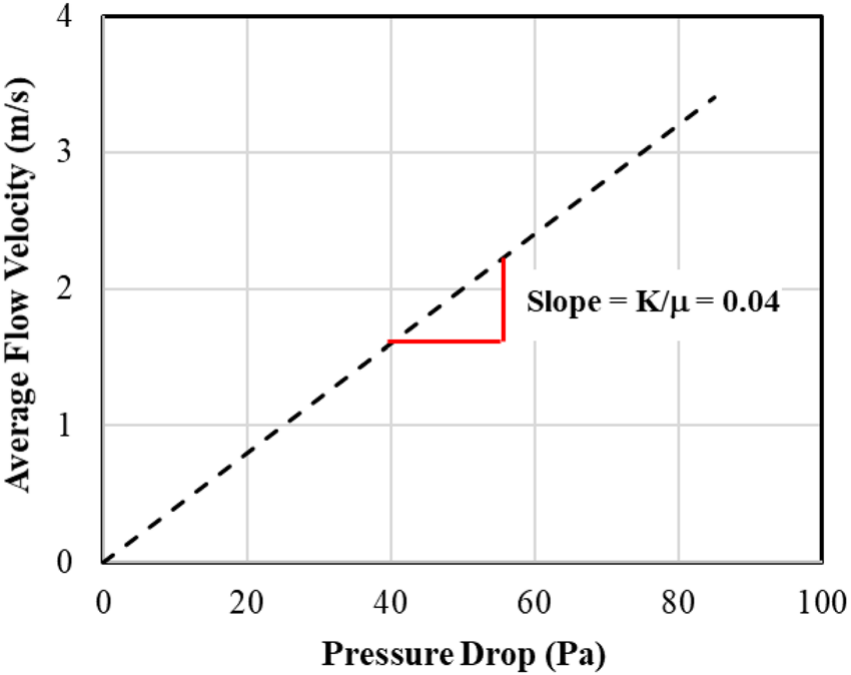
A linear relationship between pressure drop and average flow velocity in porous media.

In the Stokes flow regime, average velocity changes linearly with pressure drop, as shown in Fig. 10 as an example. The slope of this straight line is essentially the ratio of permeability of material and viscosity of fluid according to Darcy’s law (8).

The left identity right (LIR) solver^44^ (a fast and memory-efficient iterative finite volume method) was used for fluid flow simulations. The solver computes the permeability, as well as velocity and pressure fields, on 3D models. The LIR solver can be used for the numerical solution of the Stokes, Stokes-Brinkman, Navier-Stokes, and Navier-Stokes-Brinkman equations. Usually, 3D models are represented as regular voxel grids where the number of grid cells grows cubically. The LIR solver uses an adaptive grid, instead of a regular grid, to reduce the number of grid cells significantly. The basis of the adaptive grid is a data structure called LIR-tree^45^ that is used for spatial partitioning of 3D models. The pore space is coarsened in areas with small velocity and pressure variations, while keeping the original resolution near the solid surfaces and in regions where velocity or pressure vary rapidly. Pressure and velocity are discretized on staggered grids and they are arranged in such a way that each cell can satisfy the (Navier-)Stokes(-Brinkman) equations independently from its neighbor cells. Pressure variables represent an average for the whole cube and residue at the cube centers, whereas the velocities represent transport across faces between the cubes and residue on the respective faces of the cube. Two velocity variables, namely one for each neighboring cell, are introduced instead of using one velocity variable on the cell faces. The two velocity variables discretize the two one-sided limits at the center of the cell surface. The discretization of the momentum and mass conservation equations yields one linear system (block) per cell. This block structure allows using the block Gauß-Seidel algorithm as smoother in a multigrid approach as an iterative solver method.

## Conclusions

Two digital rock 3D models (ROI-1 and ROI-2) were reconstructed from ultra-high-resolution (5 nm/voxel) FIB-SEM nano-tomography image datasets of the Marcellus Shale rock sample. These 3D models were used for porosity and permeability simulations, which were conducted under five different reservoir confinement conditions (at 2,000–6,000 ft depth), in order to investigate how porosity and permeability of shales change at different depths of these unconventional reservoirs.

The two 3D models had very distinct nature in terms of their anisotropy, permeability, and confinement effects. Despite their similar porosity, the permeability of the ROI-1 was around 25 times greater than that of the ROI-2. This was due to their different connected (effective) porosities. Porosities of the non-compressed ROI-1 and ROI-2 were 13.2% and 14.7% respectively. Permeabilities of these two 3D models were calculated to be 95.6 nD (for the ROI-1), and 3.6 nD (for the ROI-2) in the Z-direction. Both 3D models were impermeable in the X-direction and there was insignificant flow in the Y-direction. This implies that permeability of the investigated shale rock sample might be dramatically different depending on the size and location of the investigated region of the sample and/or direction of flow.

Porosity reduction by approximately 3% for both 3D models (ROI-1 and ROI-2) (in relation to their original porosity) was observed under confinement at the maximum simulated depth of 6,000 ft. Permeability of the deformed ROI-1 increased by approximately 3% (in relation to its original permeability) in the Z-direction. On the other hand, permeability of the deformed ROI-2 decreased by approximately 16% (in relation to its original permeability) in the Z-direction. For these specific samples, minor effect of reservoir confinement on porosity and permeability was observed related to the pore morphology, extent of stress, and connected fluid flow pathways^45^.

## Data Availability

The data generated during and/or analyzed during the current study are available from the corresponding author on reasonable request.
